# Surgical options for male breast cancer

**DOI:** 10.1007/s10549-018-4952-2

**Published:** 2018-09-05

**Authors:** Ian S. Fentiman

**Affiliations:** Research Oncology, Guy’s Hospital, 3rd Floor Bermondsey Wing, London, SE1 9RT UK

**Keywords:** Male breast cancer, Neoadjuvant endocrine, Mastectomy, Breast conservation, Sentinel node biopsy, Reconstruction

## Abstract

**Background:**

Male breast cancer (MBC) is a rare disease for which no randomised controlled trials (RCT) have been conducted to determine optimal surgical management. The available data have been reviewed to identify reasonable options and reveal areas in need of investigation.

**Methods:**

All published series on the surgical management of MBC have been reviewed to determine approaches to treatment of the primary, the breast and the axilla together with the psychological sequelae of surgery.

**Findings:**

Mastectomy is still the major surgical offer but a convincing case can be made for the use of neoadjuvant endocrine treatment in order to facilitate breast conserving surgery. Sentinel node biopsy has been successfully used for staging MBC although nomograms for prediction of nodal status are inadequately calibrated. There are psychological sequelae of mastectomy in males and as yet no evidence that the needs of those with MBC are being met.

**Conclusions:**

Collaborative studies are required so that men can participate in meaningful RCTs to provide an evidence-based rational foundation for the surgery of MBC.

## Introduction

Men with breast cancer are a disadvantaged minority. They have been diagnosed with a disease which some considered to be an all-female affliction. This lack of awareness partly explains why more than 40% present with advanced or metastatic disease [1]. The biology of male breast cancer (MBC) differs significantly from that of female breast cancer (FBC) [2–5]. Despite this, at present, most treatment decisions are based on an extrapolation from randomised controlled trials (RCTs) in FBC. Mastectomy has been the standard surgical offer for MBC whereas breast conserving therapy is widely used for selected females with the disease and has been shown to be effective in the long-term [6–8]. For men with breast cancer, combined approaches and thoughtful surgery are needed to achieve maximal likelihood of cure together with a minimum of long-term psychological distress.

## Neoadjuvant treatment

It is extraordinary that for patients with almost invariably oestrogen receptor positive disease, often presenting with large tumours, no prospective studies of endocrine neoadjuvant therapy for MBC have been reported. Indeed, there are only a few reports of neoadjuvant chemotherapy given on an ad hoc basis [9, 10]. A potentially very useful approach has not been exploited since neoadjuvant endocrine therapy could enable some men with breast cancer to undergo a wide excision of the cancer without a need for a mastectomy or allow less extensive surgery with primary skin closure.

What form should that neoadjuvant endocrine treatment take? It has been suggested that MBC resembles post-menopausal FBC [11]. Tamoxifen has been the standard treatment for pre-menopausal women with ER+ ve breast cancer but RCTS have shown that for post-menopausal women with ER+ ve cancers, aromatase inhibitors (AIs) are superior in an adjuvant role [12]. MBC has been likened to post-menopausal FBC and so it was understandable that AIs should have also been used as adjuvant treatment for MBC [13]. Relatively small studies appeared to show a benefit from adjuvant anastrozole and letrozole [14, 15]. Subsequent larger studies were less encouraging. Harlan et al analysed outcomes in 512 MBC cases derived from the surveillance, epidemiology and end-results (SEER) database [16]. Of these, 124 (28%) received adjuvant hormonal therapy (tamoxifen 95, AI 19, tamoxifen+ AI 8, other 2). Although there was a significant reduction in cancer mortality among those given tamoxifen (HR 0.04, CI 0.1, 0.99) compared with no systemic therapy, adjuvant AIs did not reduce deaths (HR 1.2, 95% CI 0.4, 3.8).

Eggermann et al studied 257 MBC patients with ER+ ve disease reported to German cancer registries and compared their outcome with 2785 FBC cases all of whom received endocrine therapy [17]. The median follow-up of was 106 months for females and 42 months for males. Cases were matched for age, tumour stage, tumour grade, nodal status, HER2 status and receipt of chemotherapy in a 2:1 F/M ratio. The female and male patients were matched 2:1. Tamoxifen was given to 316 women and 158 men and AIs to 60 and 30 respectively. TAM-treated patients of both genders had similar 5-year OS but FBC patients treated with AIs had significantly better 5-year OS (85.0%) compared with AI-treated MBC cases 85% versus 73.3% (*p* = 028). The probable explanation is that testicular production of oestrogen (approximately 20%) is not abolished by AIs [18]. This indicates that tamoxifen should be the neoadjuvant endocrine therapy of choice in MBC.

Unfortunately there can be a problem with patient compliance in men taking tamoxifen. Annelli et al investigated the side effects of adjuvant tamoxifen treatment in 24 MBC cases of whom 15 (63%) complained of one or more side effect [19]. Of these, 19 had ER− ve primary tumours and the men were treated from 1990 to 1993. These included reduced libido (7) weight gain (6), hot flushes (5) and altered mood (5). One developed deep vein thrombosis. In toto, 5 (21%) stopped taking tamoxifen within 1 year compared with a female discontinuation rate of 10% [20]. Similar findings were reported by the Ottawa Hospital Cancer Centre between with 50% suffering side effects and 24% stopping the treatment in one case because of a pulmonary embolism [15]. Failure to take adjuvant therapy can lead to serious consequences. Xu et al reported a cohort of 116 MBC patients with ER+ ve disease [21]. After 1 year only 65% were still taking tamoxifen, 46% after 2 years, 29% at 3 years, 26% at 4 years and only 18% in the final year. The 10-year disease-free survival of the compliant patients was 96% compared with 42% in the non-compliers. If used as neoadjuvant treatment for shorter durations compliance might be less of a problem.

Other potential approaches include the use of GnRH analogues such as goserelin which reversibly achieve a testicular ablation but such treatment may not be acceptable to many men with MBC. The historical treatment for advanced or metastatic MBC was surgical orchidectomy but this was rejected by more than 50% of patients [22]. Balancing efficacy and toxicity will need international cooperation to run randomised controlled trials (RCTs) of neoadjuvant endocrine therapy for MBC. Collaboration has already started but until appropriately powered studies have been conducted, including quality of life metrics, treatment will continue to be given on an empirical basis without an evidence base [23–25].

## Breast conserving surgery versus mastectomy

Unlike the situation in female breast cancer, there are no RCTs to confirm the safety of breast conserving surgery for selected cases of MBC [26–28]. Such evidence as there is derives from historical comparisons which have the disadvantage of the associated unknown selection biases. In a series of 257 Danish MBC cases treated between 1943 and 1972, 78% had operable disease but only 15 (8%) had local excision [29]. Guinee et al reported a cohort of 308 MBC cases with operable disease of whom 30 (10%) underwent breast conserving surgery [30]. Of 229 Canadian cases, 168 were treated by mastectomy and 8 (3.5%) had a local excision and axillary clearance [31]. In the large French cohort of 489 cases only 42 (8.6%) had breast conserving surgery [32].

The surveillance, epidemiology and end-results (SEER) database has been extensively interrogated with regard to MBC. Cloyd et al reported that of 5425 males treated between 1983 and 2009, 4707 (87%) underwent mastectomy and 718 (13%) had lumpectomy, increasing from 11% between 1983 and 1986 to 15% in 2007–2009 [33]. Ten-year breast cancer-specific survival was 83% after lumpectomy and 77% following mastectomy.

In a stage-specific analysis of 4276 cases diagnosed between 1973 and 2008 Fields et al reported breast conserving surgery was used in only 10% [34]. There was similar cancer-specific survival in men treated by lumpectomy and radiotherapy compared with mastectomy (hazard ratio 1.33; 95% CI 0.49–3.61; *P* = 0.57). Leone et al investigated the relationship between clinico-pathological variables, locoregional treatment and overall survival (OS) in 1283 men with T1a,b,c,N0, M0 disease registered with SEER between 1988 and 2012 [35]. The sub-stages were: T1a 7%, T1b 21% and T1c 72% and within each of these mastectomy was performed in > 74%. There was no significant difference in OS for those treated by breast conservation or mastectomy. Risk factors for worse survival were older age, higher grade tumours, no surgery, no axillary staging and being unmarried.

Zaenger et al examined outcomes for 1777 males with T1/2, N0 disease, treated between 1998 and 2011 [36]. Most were treated by radical or simple mastectomy, with or without post-operative radiotherapy. Only 296 (17%) had breast conserving surgery with 135 (46%) receiving post-operative radiotherapy. The actuarial 5-year cancer-specific survival was 100% for the BCT group and 97% for MRM, for stage 1 and 91.2% for stage 2. This needs to be interpreted with caution because of the relatively short duration of follow-up.

In a study of 42 MBC cases treated in Massachusetts between 1990 and 2003, 30 underwent modified radical mastectomy (MRM), 4 simple mastectomy (SM) and 8 had breast conserving surgery (BCS) [37]. A multidisciplinary group assessed musculoskeletal function including arm oedema and range of shoulder movement. Table 1 shows that there was reduced morbidity after BCS with no lymphoedema or limitation of shoulder movement.

**Table 1:** Morbidity after surgery for MBC (Fogh et al. [37])

Procedure	Lymphoedema	Shoulder restriction
MRM (*n* = 30)	7 (23%)	8 (27%)
TM (*n* = 4)	0	2 (50%)
BCS (*n* = 8)	0	0

## Psychological sequelae

Unsurprisingly there are relatively limited data concerning the psychological consequences of the diagnosis and treatment of MBC. Anxiety/depression, body image, cancer-specific distress and coping capacity were determined by Brain et al using a cross-sectional questionnaire on 161 MBC cases [38]. Clinically treatable levels of anxiety and depression were present in 6% and 1% respectively which is substantially lower than that reported in FBC [39]. Nevertheless, high levels of cancer-specific distress were reported by 23%. Within the US 2009 Behavioural Risk Factor Surveillance System (BRFSS) survey there were 66 MBC cases and Andrykowski compared them with 198 age-matched cancer-free control males [40]. The MBC cases had a significantly increased risk of obesity, comorbidity, reduced activity, poorer life satisfaction and worse general health.

Kowalski et al examined health-related quality of life (HRQoL) in 84 male breast cancer patients and compared HRQoL scores with FBC and male non-MBC populations. In relation to FBC patients, men with MBC had higher scores in 7/8 subscales physical functioning, role functioning-physical and emotional, bodily pain, vitality, social functioning and mental health. In contrast, when compared with the general male population, MBC patients showed major defects in emotional and physical role functioning. Gaitanidis et al examined the SEER database from years 1973–2013 to determine the rate of suicide and risk factors in breast cancer patients. Of 474,128 patients 773 (0.16%) had killed themselves. The significant risk factors were age < 30 years, male sex and single status, particularly in the first year after diagnosis.

## Sentinel node biopsy

There have been several series reporting ≥ 30 sentinel node biopsies for MBC, all from the USA which have been summarised in Table 2 [41–46]. The lowest identification rate (90%) occurred in the series in which not all cases received both isosulfan blue and technetium-99m [35]. This suggests that whenever possible a joint identification approach should be used.

**Table 2:** Results of sentinel node biopsy in MBC

Author	*N*	Technique	Identification (%)	Node positive (%)
Boughey et al. [41]	30	IB & Tc	100	37
Rusby et al. [42]	31	IB/Tc 16 IB 5 Tc 10	90	55
Gentilini et al. [43]	32	Tc	100	19
Flynn et al. [45]	78	IB & Tc	97	49
Kiluk et al. [44]	34	IB & Tc	100	29
Maraz et al. [46]	25	IB & Tc	100	48

*IB* Isosulfan blue, *Tc* Technetium-99m

Vaysse et al examined whether predictive factors for axillary nodal status derived from FBC were applicable to MBC [47]. They used 2 nomograms: Institut Curie (IC) [48] and Memorial Sloan-Kettering (MSKCC) [49]. The calibration and discrimination performance of both nomograms were tested in 80 men with operable cancer. Axillary lymph node involvement was present in 37 (46%). The area under the curve (AUC) of IC and MSKCC was 0.66 and 0.64 respectively, indicating inadequate calibration of both. This could have been the result of a relatively small sample size or possibly different biological determinants in the 2 genders.

## Reconstruction

Until recently, reconstruction with skin flaps has used solely to achieve skin closure after mastectomy for MBC but no large series have been reported. Chastel et al used Limberg flaps for two males following modified radical mastectomy with a satisfactory result [50]. It has been argued by Spear and Bowen that a transverse rectus abdominis (TRAM) flap not only replaces the skin and fat but also provides hair-bearing cover similar to the normal male breast skin [51]. Others have also the robustness of TRAM flaps even after local relapse of MBC [52, 53]. Another approach in a debilitated patient needing a mastectomy with a chest wall defect is the delto-pectoral flap (DP) flap [54].

The latissimus dorsi (LD) flap is the reconstruction workhorse in females undergoing mastectomy and reconstruction and this technique has been used successfully in males with large and borderline operable breast cancer [55, 56]. One disadvantage is that LD flaps reduce shoulder function. This long-term morbidity may have to be accepted if local control of the cancer is to be achieved.

A variety of oncoplastic techniques have evolved to achieve a better and more symmetrical outcome after breast conserving surgery. These may be applicable for men who have both MBC and gynaecomastia, so that the cancer can be excised with a better cosmetic outcome and the contralateral breast made symmetric by reduction mammoplasty [57].

## Ductal carcinoma in situ

Men have only partly escaped the epidemic of DCIS which manifests as microcalcification and represents 20% of the cancers picked up by screening. As was the case before the introduction of mammographic screening some are symptomatic. When MBC and FBC were compared using a SEER database, DCIS comprised 280/2984 (9.4%) of male cases and 53,928/454,405 (11.9%) of FBC [58]. In a series of 84 pure DCIS male cases in the Armed Forces Institute of Pathology specimen archive the median age at diagnosis was 65 years [59]. Most presented with a lump (58%) and bloody nipple manifested in 35%. In three histological studies the papillary subtype was the commonest, followed by mixed papillary and cribriform [48, 49, 60]. No pure DCIS specimens were high grade disease, with 57% low and 43% of intermediate grade.

Surgical decisions as in so many aspects of MBC have been made on an *ad hoc* basis. As an example, in a SEER series of 512 MBC cases there were 58 with DCIS [16]. Of these, 38 (66%) were treated by mastectomy, and one also was given post-operative radiation. Nipple-conserving surgery was performed in 18 cases of whom 7 received breast irradiation. Two cases had no surgery and the majority, 70%, had no axillary surgery. When the axilla was explored 8 had a full dissection and 9 had sentinel node biopsy. Six cases received adjuvant tamoxifen but no data were available concerning outcome. In the twenty-first century men with DCIS should have at most a sentinel node biopsy and be spared an automatic axillary clearance.

## Conclusions

The rarity of MBC is the reason why, whenever possible, all cases enter collaborative studies which will include the opportunity to participate in meaningful RCTs. As a model of successful clinical research collaboration the Danish Breast Cancer Cooperative Group (DBCG) has conducted landmark RCTs in a country with only 5½ million inhabitants [61]. In countries with National Health services such as the United Kingdom, networks based around hubs of expertise could be set up. In the UK, with 350 new cases of MBC every year, 3 hubs would each oversee > 100 cases annually. Patients would not need to travel to the hub. Cases would be discussed by a central multidisciplinary meeting together with a senior clinician from referring hospital using telephone conferencing. Those men agreeing to participate would be able to enter National/International RCTs.

Information needs of new cases could be met by an outreach services provided by appropriately trained Breast Care Nurses with support from selected MBC patients, in their homes or at the local hospital. A major step towards reassuring worried patients would be the knowledge that they were being cared for by experienced professionals. The hub team would ensure central registration of all MBC together with central histopathological review to collect a minimum data set so that epidemiological studies could be rendered more effective.
